# Recreational Boats and Turtles: Behavioral Mismatches Result in High Rates of Injury

**DOI:** 10.1371/journal.pone.0082370

**Published:** 2013-12-11

**Authors:** Lori A. Lester, Harold W. Avery, Andrew S. Harrison, Edward A. Standora

**Affiliations:** 1 Department of Agriculture and Natural Resources, Delaware State University, Dover, Delaware, United States of America; 2 Department of Biology, Drexel University, Philadelphia, Pennsylvania, United States of America; 3 The Leatherback Trust, Haddonfield, New Jersey, United States of America; 4 Department of Biology, Buffalo State College, Buffalo, New York, United States of America; 5 Williamsville Central Schools, Williamsville, New York, United States of America; Monash University, Australia

## Abstract

Recreational boats are a dominant feature of estuarine waters in the United States. Boat strike injury and mortality may have a detrimental effect on populations of diamondback terrapins (*Malaclemys terrapin*), a keystone species in estuarine ecosystems. In Barnegat Bay, New Jersey, 11% of terrapins (n = 2,644) have scars consistent with injuries from boats. Conservative estimates of injury rates from boats increased from 2006 to 2011. When exposed to playback recordings of approaching boat engines of varying sizes and speeds *in situ*, terrapins did not significantly change their behavior in response to sounds of boat engines of different sizes. The lack of behavioral response of terrapins to boat sounds helps explain high rates of injury and mortality of terrapins and may threaten the viability of terrapin populations. Boater education courses that discuss impacts of boats to wildlife, combined with closure of areas of high terrapin densities to boating, are necessary to protect terrapins and other aquatic species from injury and mortality caused by motorized boats.

## Introduction

Recreational boating is a popular pastime in the USA where there are over 12 million registered boats [1]. Recreational boats may affect aquatic animals directly by causing injury or mortality and indirectly through behavioral or physiological responses to anthropogenic sounds. Many aquatic species are directly affected by recreational boat propeller strikes including crocodiles [2], turtles [3], birds [4], and marine mammals [5]. Behavioral and physiological responses or the lack thereof of aquatic animals to boat sounds may lead to reduced fitness by lowering survival rates and/or reproductive rates [6], [7].

Many turtles can detect sounds under 1000 Hz including estuarine [8], [9], freshwater [10], [11], and sea turtle species [12]. Recreational boats produce low-frequency sounds that overlap with turtle hearing ranges [13]. Therefore, it is logical to conclude that turtles can hear and possibly avoid oncoming boats. However, some populations of diamondback terrapins (*Malaclemys terrapin*) along the Atlantic and Gulf coasts of the United States experience high (6 to 20%) rates of injuries from anthropogenic sources [3], [14]–[16]. Thus, terrapins may not respond behaviorally to avoid boats. Boat injuries also reduce body condition of male terrapins and survivorship of both male and female terrapins [3]. Mortality can also occur via blunt-force trauma from being hit by a boat or lethal injury from a propeller [17].

Loggerhead (*Caretta caretta*) and green (*Chelonian mydas*) turtles respond to anthropogenic boat sounds by increasing submergence time between breaths, spending more time underwater, and swimming to the surface [18], [19]. However, these studies were performed in laboratory aquaria [19], [20] where sounds are distorted due to reverberation and resonance [21]. Some sea turtle species respond to seismic air guns *in situ* with erratic behavior but results from these studies are compromised by small sample sizes and individual differences in behavioral response [22], [23]. It is unknown whether free-living diamondback terrapins respond to the sound of oncoming boats.

Motorized boat densities in estuaries are among the highest of any aquatic ecosystem in the world [1] and the diamondback terrapin is the only turtle species in the USA to inhabitat brackish habitats exclusively throughout its life. Therefore, boats are a potentially important threat to terrapins and it is important to determine how terrapins respond to boat sounds. The goals of this study were to (1) measure the rate of terrapin injury due to boat strikes in Barnegat Bay, NJ in order to determine the direct impact of boats on the terrapin population, and (2) determine whether terrapins behaviorally respond to boat engine sounds *in situ*. We recorded injury data for wild terrapins captured over seven years in a mark-recapture population study. We then used recorded underwater sounds of different sized boat engines to determine the behavioral responses of terrapins to these recordings. Our study was performed *in situ* to better understand how turtles respond to sounds in their natural environment.

## Materials and Methods

### Ethics Statement

All research protocols were approved by the Institutional Animal Care and Use Committee at Drexel University (protocol #18296). Special Use Permit (#10038) was provided by the Edwin B. Forsythe Wildlife Refuge and Scientific Holding (#2010105) and Collecting (#29102) Permits were provided by the New Jersey Division of Fish and Wildlife and issued to HWA.

### Study Site

Our study was conducted in the Barnegat Division of the Edwin B. Forsythe National Wildlife Refuge (Forsythe) in the Barnegat Bay estuary. Barnegat Bay is a 70 km long estuary located along the eastern coast of New Jersey, USA and is adversely affected by many anthropogenic factors, including high levels of recreational boating [24].

### Field Sampling Techniques

**Figure 1:**
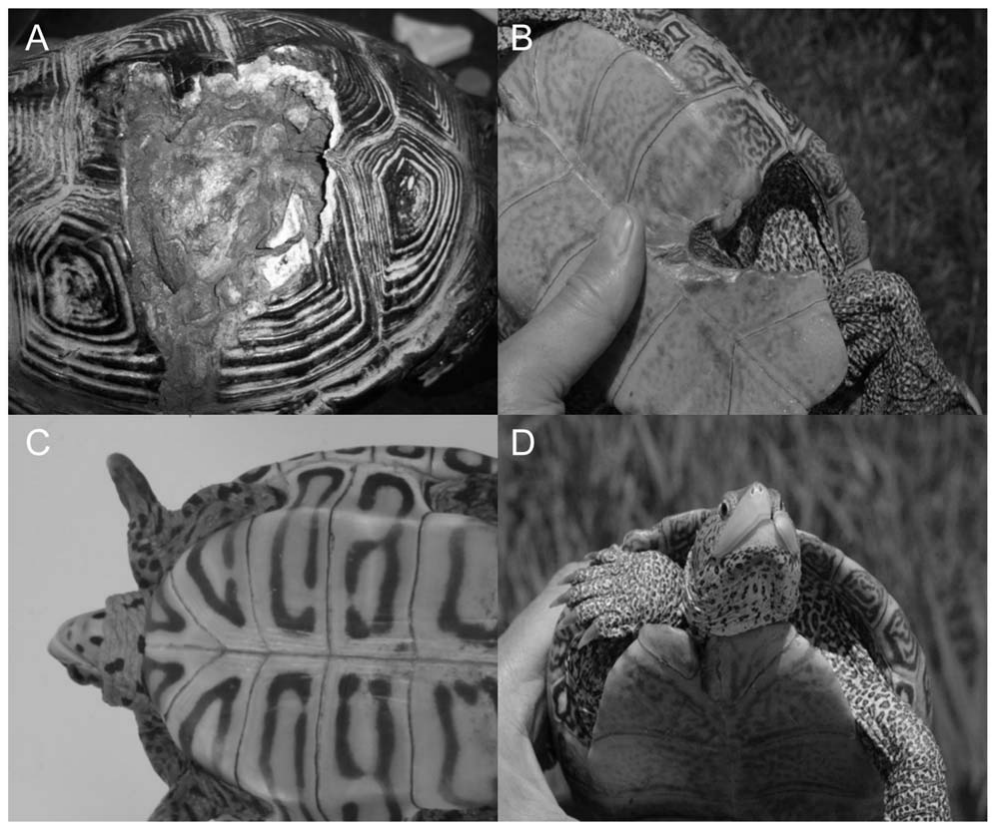
Anthropogenic injuries to diamondback terrapins. Diamondback terrapins were classified as having an anthropogenic injury if damage occurred to two or more vertebral or costal scutes (A), two or more plastral scutes (B), and/or three or more marginal scutes. Photo A shows a female terrapin with an anthropogenic injury to two vertebral and two costal scutes whereas photo B shows an anthropogenic injury to two plastral scutes. Many injured terrapins also had missing limbs (C), tail, or head (D) injuries. Photo C shows a terrapin missing its front left limb which could be due to a boat injury or a predator. In photo D, the terrapin has an anthropogenic injury to its beak and anterior plastron. This injury was assumed to be from a boat because it appeared to be a slash from a propeller that occurred from the plastron through the beak to the anterior carapace. Anthropogenic injury rates are likely an underestimate of the actual number of terrapins hit by boats and automobiles because many of these injuries lead to mortality.

We captured diamondback terrapins using hoop nets, fyke nets, dip nets, and by hand as part of a long term population study of the terrapins in Barnegat Bay from 2006 to 2011. We recorded location of injury including carapace, plastron, bridge, tail, limb, and head (Fig. 1). For each terrapin capture, we described shell injuries by recording position and names of broken scutes, and by drawing injuries on a diagram of the terrapin shell. We assumed that major shell damage (defined as injury to two or more adjacent vertebral, costal, or plastral scutes, or three or more adjacent marginal scutes) to adult turtles was caused by boats [3]. We assumed that major shell injuries were from recreational boat propellers because terrapin nesting beaches are not located near roads in Forsythe. In general, diamondback terrapins with boat injuries tend to have scars from propellers and those with automobile injuries tend to have crushed shells. Some diamondback terrapins in Forsythe Refuge appear to be hit by the hull of boat or personal watercraft (PWC) instead of the propeller and display a crushed carapace similar to those injuries seen in terrapins that have been hit by automobiles. We used linear regression to determine if there was a temporal change in injury rates and to determine if larger terrapins were more likely to be injured by a boat than smaller individuals over a six year period.

### Experimental Methodology

We exposed small (n = 40, 400 to 600 g body mass) and large (n = 40, 1,000 to 1,200 g body mass) non-gravid, uninjured female terrapins to playback recordings of approaching boat engines. Female terrapins were selected because they attain significantly larger body size than males allowing total mass of data loggers attached to terrapins to be under 5% of body mass [25]. Mean mass of captured male terrapins was 266.1 g±50.1 SD, which is too small for data logger attachment. Non-gravid females were selected because they were less likely to exit water to pursue nesting areas. We used small and large size classes of female terrapins because older (i.e., larger) females may have reduced hearing capability compared to younger (i.e., smaller) females.

### Playback Recordings

We recorded sounds of four different recreational boats varying in length and outboard engine size with a digital recording computer (Sound DSA ST 191; Cetacean Research Technology; Seattle, WA, USA) and a hydrophone (C54XRS; Cetacean Research Technology; Seattle, WA, USA). Boats included a Lowe boat (Johnson 9.9 hp outboard two cycle motor, 4.3 m length, 22.9 km/hr speed), a Polar Kraft boat (Mercury 25 hp outboard two cycle motor, 4.3 m length, 41.9 km/hr speed), an Action Craft boat (Johnson 110 hp outboard two cycle motor, 5.5 m length, 40 km/hr speed), and a Parker boat (two Johnson 150 hp outboard four cycle motors, 8.5 m length, 53.4 km/hr speed). Each of the four boats was driven at maximum speed past the hydrophone parallel to shore within 1 m from the hydrophone. We measured the sound spectrum of each boat with SpectraPRO 3.32 (Cetacean Research Technology; Seattle, WA, USA) for each 1 min long recording [26].

### Data Loggers and Transmitters

**Figure 2:**
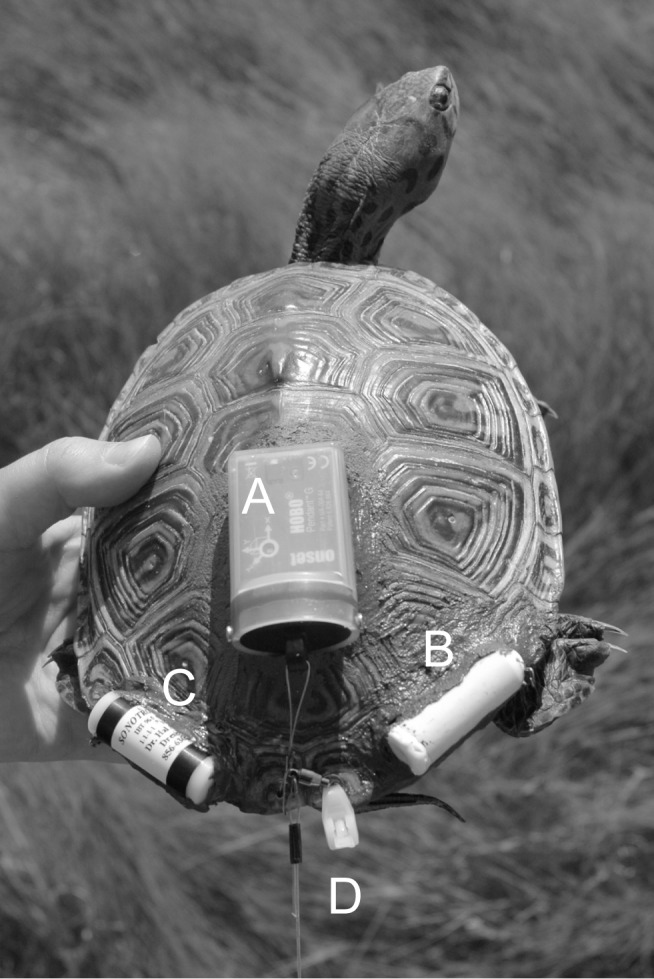
Data loggers and transmitters attached to an adult female diamondback terrapin. A HOBO Pendant G data logger recorded the orientation of the terrapin in the water every 1(A). A Data Storage Tag milli-L temperature and depth data logger recorded depth of the terrapin every 1 s (B). Sonic (C) and radio (D) transmitters allowed us to relocate terrapin in case of escape. Transmitters and data loggers weighed <5% body mass of terrapins.

To measure changes in swimming behavior in relation to boat sounds, we outfitted each terrapin with a HOBO Pendant G acceleration data logger (UA-004-64, accuracy ±2.5°; Onset Computers; Bourne, MA, USA) and a Data Storage Tag (DST) milli-L temperature and depth data logger (depth range 10 cm to 20 m, depth accuracy ±8 cm; Star-Oddi; Reykjavik, Iceland; Fig. 2). The HOBO pendant G data logger recorded x-, y-, and z-axis orientation of the terrapin in degrees every 1 s and the DST recorded depth and temperature every 1 s during the trials. We calculated mean change in pitch (x-axis) and roll (y-axis) of terrapins before, during, and after sound by taking the mean of the absolute values of each value minus the value from the previous 1 s.

A sonic transmitter (IBT-96-5; 40 kHz; 8.5 g; Sonotronics; Tucson, AZ, USA) and a radio transmitter (V2B154; two-stage; 164 MHz; 27 g; Sirtrak; Havelock North, New Zealand) enabled relocation of the terrapin during the study (Fig. 2). The radio transmitter was embedded in a streamlined syntactic foam float tethered to the posterior marginal scute of the terrapin to allow continuous reception of radio signals. The radio transmitter float also allowed us to visualize where the terrapin was swimming during trials. We detected sonic transmitters using a submersible sonic receiver (N15A235B; Dukane Underwater Acoustic Location Receiver; St. Charles, IL, USA) and radio signals were detected with a two-element yagi antenna connected to a radio receiver (R1000; Communications Specialists Incorporated; Orange, CA, USA).

### Experimental Design

We exposed each terrapin to playback recordings in a 60 m segment of a canal (locally called a mosquito ditch) that was approximately 1.5 m deep by 2 m wide located in Forsythe. Mosquito ditches are straight, narrow canals that are dug to control mosquito populations. We blocked off both ends of the mosquito ditch with plywood to ensure that terrapins were in the canal and tidal water flow that could otherwise influence behavior was minimized. All trials were completed within two hours of high tide to ensure the canal had maximum water depth (∼1.5 m).

An underwater speaker (LL9816; Lubell Labs; Columbus, OH, USA) was suspended at a depth of 75 cm at the midpoint of the 60 m long canal for playing back recorded boat sounds. The speaker was connected to a Speco Amplifier (PAT 20 TB 20 Watt 12 V PA; Speco Technologies; Amityville, NY, USA) which broadcasted boat motor sounds played back with the computer program, SpectraPRO 3.32. We monitored playback recordings prior to each trial with the hydrophone (C54XRS) suspended at various distances (1 m, 5 m, and 10 m) from the underwater speaker to determine sound propagation in the canal.

We initiated each trial by releasing a terrapin into the water at a randomly chosen end of the canal and allowed it to acclimatize for fifteen minutes. The terrapin was allowed to swim freely and when its radio transmitter float was 10 m from the speaker, we started a playback recording of a boat motor. Each trial ended when the terrapin completed swimming a total of 60 m regardless of whether it swam straight or turned during the trial. We determined swimming speed by timing how long it took each terrapin's radio transmitter float to travel through each 10 m section. We standardized swim speed as a function of body length of each terrapin using straight carapace length (expressed as body lengths s^−1^). Six trials were completed per terrapin: three were sound trials where one of the four boat engine recordings was played and three were control trials were the terrapin was allowed to swim past the underwater speaker with no sound playing.

### Data Analyses

We used a multivariate linear mixed-effects model to test for significant behavioral response variables (i.e., swimming speed, swimming depth, change in pitch, and change in roll) before, during, and after exposure to playback recordings. Fixed effects included treatment (sound or control), terrapin size (small or large), and time (before, during, or after sound playback). Behavioral measurements were repeated three times for each individual terrapin. We used a nested design to avoid pseudoreplication in the analysis. The within-individual repeated measurements were added as random effects including individual terrapin (n = 10) and number of trials (three trials per terrapin). We used restricted maximum likelihood (REML) to model changes in terrapin behavior in response to playback sound with the package lme4 [27] in program R 2.14.0. P-values were obtained by likelihood ratio tests of the full model against the model without the effect of sound exposure.

## Results

**Figure 3:**
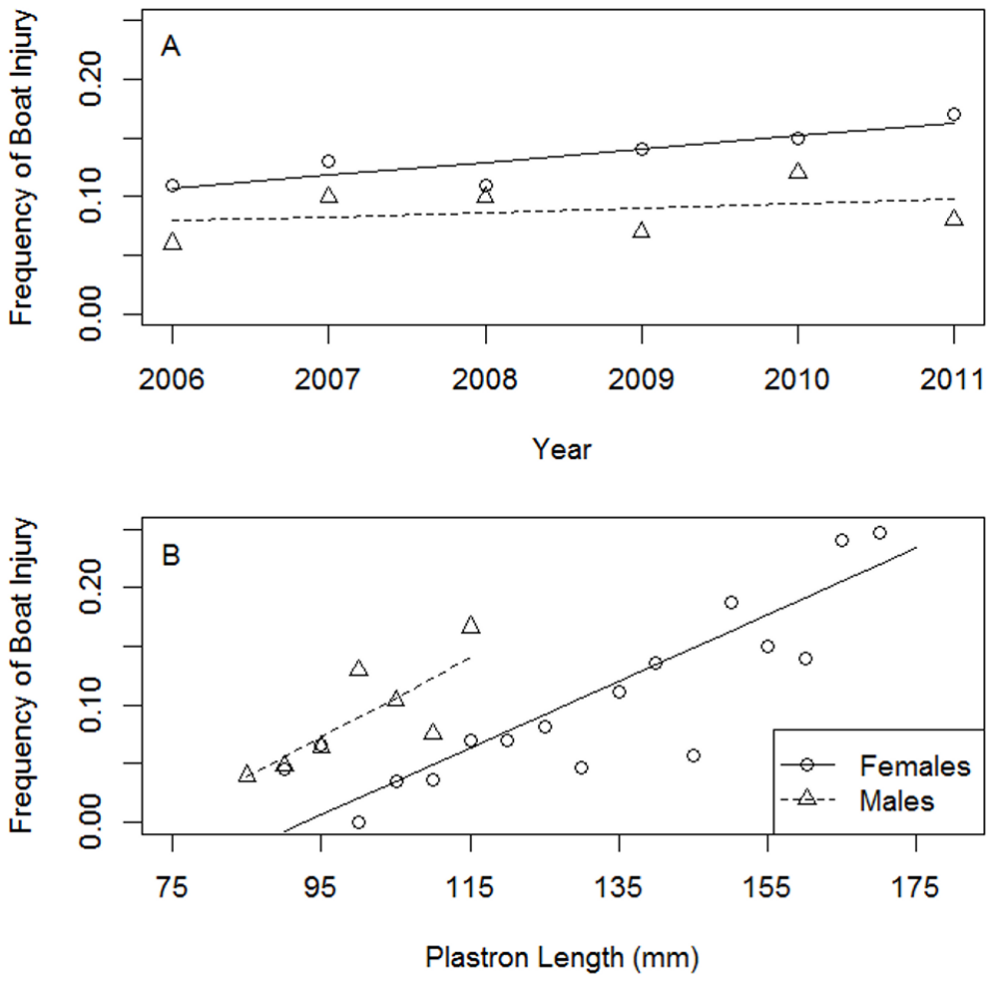
Injury rates of diamondback terrapins in Barnegat Bay, New Jersey. Mean boat injury frequency increased for adult female terrapins captured in the Edwin B. Forsythe Wildlife Refuge from 2006 to 2011 (A; linear regression, Y = 0.01 X -22.2, R^2^ = 0.74, P = 0.02). Large female (Y = 0.003 X – 0.3, R^2^ = 0.73, P<0.001) and male (Y = 0.003 X – 0.2, R^2^ = 0.56, P = 0.03) terrapins were more likely to be injured by a boat than smaller individuals (B). The number of boat injuries resulting in death was unknown because dead animals were lost to the natural system.

Eleven percent of diamondback terrapins in Edwin B. Forsythe Wildlife Refuge had substantial boat injuries (Fig. 3; n = 291 of 2,644). Mean rates of boat injury for adult female terrapins increased significantly from 2006 to 2011 (Fig. 3A; Y = 0.01 X – 22.2, R^2^ = 0.74, P = 0.02). However, mean rates of boat injury for male terrapins did not increase over time (Y = 0.004 X – 7.4, R^2^ = 0.10, P = 0.55). Large female (Y = 0.003 X – 0.3. R^2^ = 0.73, P<0.0001) and male (Y = 0.003 X – 0.2, R^2^ = 0.56, P = 0.03) terrapins are more prone to boat injuries than smaller individuals of the same sex (Fig. 3B).

**Figure 4:**
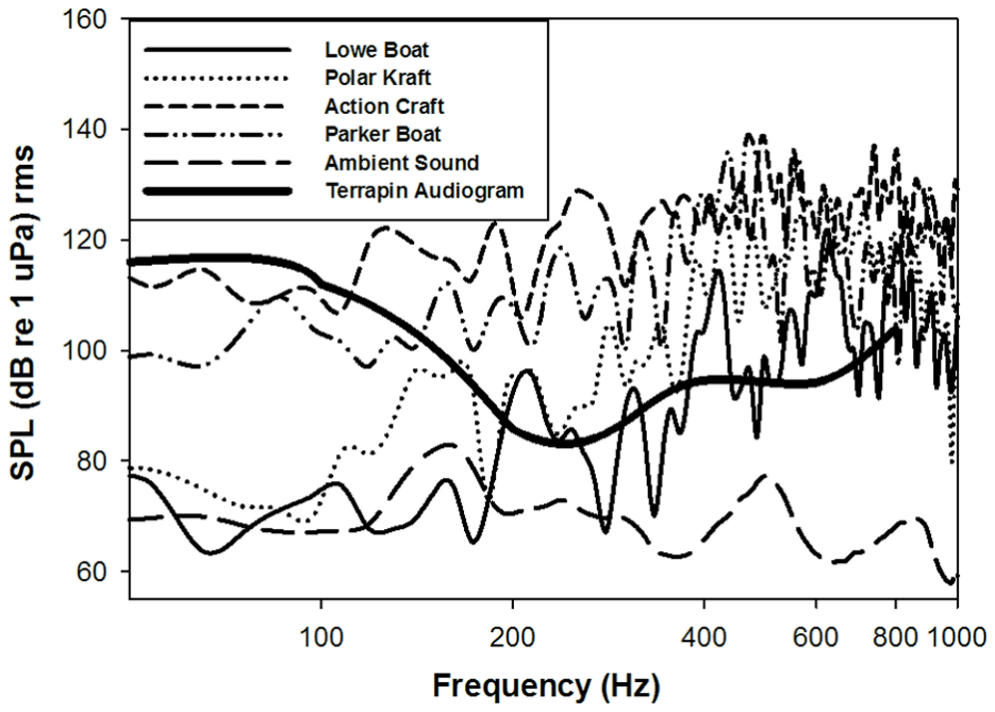
Mean boat spectrums. Each terrapin was exposed to one of four different boat engine recordings: Lowe boat (9.9 hp motor), Polar Kraft boat (25 hp motor), Action Craft boat (110 hp motor), and Parker boat (two 150 hp motors). The maximum sound pressure level (SPL) recorded from each boat varied from 100 to 140 dB re 1 µPa in the 400 to 600 Hz range. The range of best hearing for terrapins (i.e., the frequencies at which terrapins can hear the lowest thresholds) underwater is also from 400 to 600 Hz suggesting that terrapins should be able to hear the boat recordings [9].

**Figure 5:**
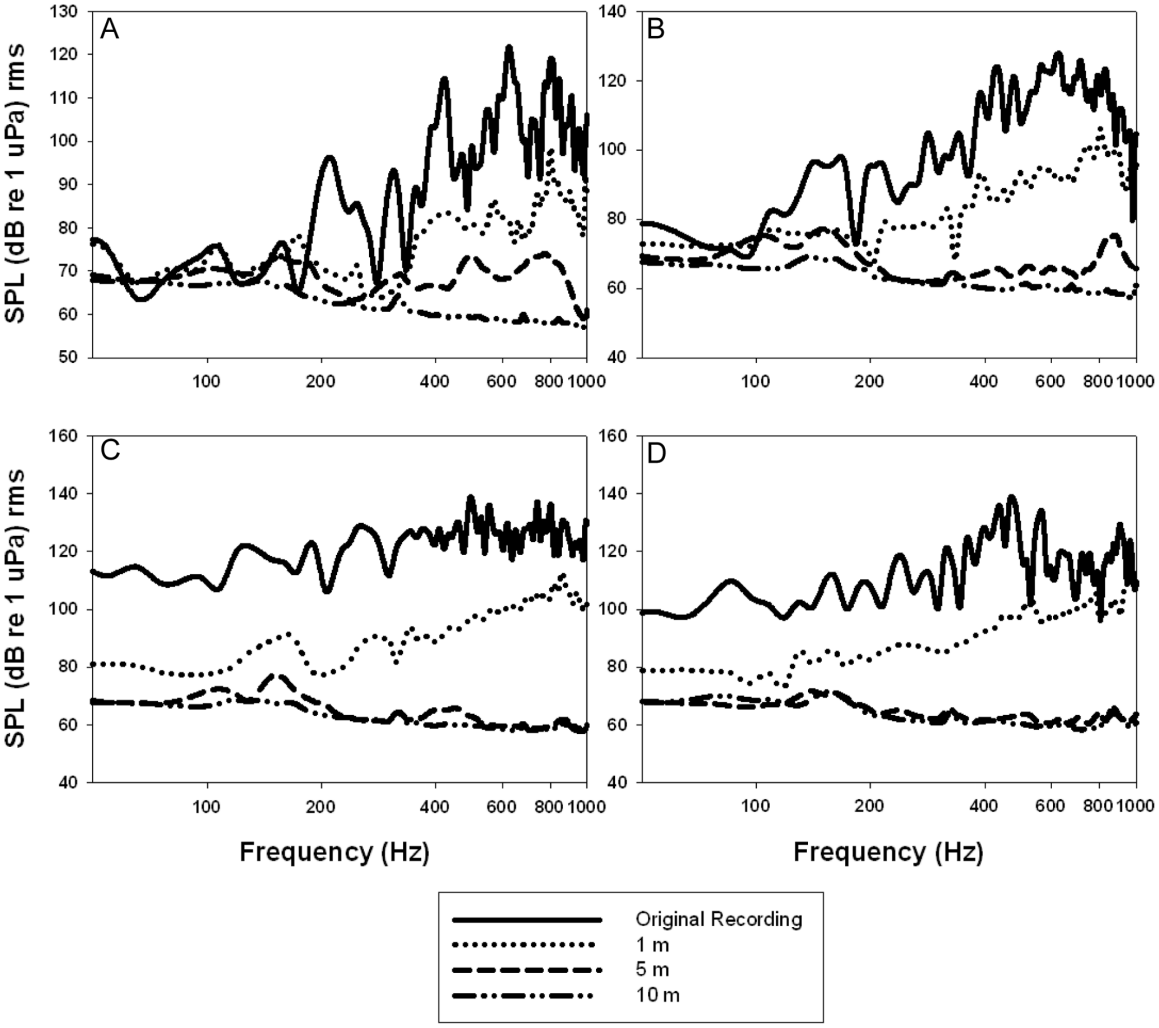
Sound propagation in the experimental canal. We measured mean spectrums of each boat recording (A – Lowe Boat, B – Polar Kraft, C – Action Craft, D – Parker Boat) at various distances (1 m, 5 m, and 10 m) from the underwater speaker. At 1 m from the speaker, the playback boat sound was a mean SPL of 15 to 28 dB re 1 µPa less than original recording depending on which boat sound was playing. At 5 m from speaker, the playback boat sound was not detectable from ambient sound in the canal probably because the sound attenuated rapidly due to the shallow water and soft mud substrate on the sides and bottom of the mosquito ditch.

Boat engine sounds in Barnegat Bay were in the hearing range of terrapins [8] with low-frequency components with maximum sound pressure levels (SPL) between 100 and 140 dB re 1 µPa rms in the 400 to 600 Hz range (Fig. 4). When we measured spectrums of the playback recordings at 1 m from the underwater speaker, playback boat sounds had SPLs that were lower than the corresponding original recording but were still in the hearing range of diamondback terrapins (Fig. 5). At 1 m from speaker, mean SPL was 15 dB re 1 µPa rms lower than the original recording for Lowe Boat, 18 dB re 1 µPa rms for Polar Kraft, 28 re 1 µPa rms for Action Craft, and 20 re 1 µPa rms for Parker Boat. When sound was measured 10 m from the underwater speaker, the playback boat sound was no longer detectable from ambient sound in the mosquito ditch due to the shallow water causing attenuation of the sound. Thus terrapins may not be able to distinguish the boat sound from the ambient sound in the mosquito ditch when located more than 10 m from the speaker.

Terrapins did not significantly change behavior in response to playback recordings. Swimming speed did not differ before, during, or after playback recordings (Table 1 & 2; p-values from 0.14 to 0.76). Mean swimming depth of terrapins varied from 0.1 m to 0.25 m but did not significantly change in response to exposure to acoustic recordings of approaching boat engines (Table 2 & 3; p-values from 0.21 to 0.81). Pitch and roll varied from 3° to 5°. There were no significant differences in mean absolute value of change in pitch or roll in response to playback recordings in either size class (Table 2 & 4; p-values from 0.07 to 0.70 for pitch and 0.09 to 0.73 for roll) indicating that terrapins were not making sudden or erratic movements in response to boat sounds. Since the effects of terrapin size and boat engine size on behavioral response variables were not statistically significant, analyses were redone with these fixed effect variables removed. Diamondback terrapins still did not significantly change behavior in response to playback recordings of boat engine sounds (p-values from 0.12 to 0.92).

**Table 1:** Mean swimming speed of diamondback terrapins in response to playback recordings of approaching boats.

			Swimming Speed (body lengths/s)					
Motor Size (hp)	Trial Type	Body Size	25 m	15 m	5 m	5 m	15 m	25 m
9.9	Sound	Large	1.68±0.05	1.69±0.06	1.90±0.11	2.22±0.09	1.97±0.06	1.80±0.05
	Control	Large	1.60±0.08	1.88±0.09	1.87±0.05	2.08±0.08	1.95±0.07	1.65±0.07
	Sound	Small	1.61±0.07	1.79±0.06	1.84±0.16	2.13±0.19	1.89±0.09	1.83±0.12
	Control	Small	1.74±0.07	1.93±0.09	1.77±0.07	1.91±0.06	1.74±0.05	1.67±0.09
25	Sound	Large	1.64±0.06	1.60±0.08	1.89±0.11	1.93±0.08	1.88±0.06	1.73±0.10
	Control	Large	1.81±0.07	1.87±0.07	1.79±0.06	1.80±0.05	1.77±0.05	1.63±0.06
	Sound	Small	1.79±0.08	1.66±0.05	1.79±0.07	1.77±0.08	1.77±0.10	1.66±0.07
	Control	Small	1.78±0.10	1.73 0.06	1.80±0.09	1.85±0.12	1.84±0.07	1.79±0.19
110	Sound	Large	1.91±0.09	1.80±0.06	2.02±0.10	2.00±0.10	1.93±0.07	1.82±0.04
	Control	Large	2.00±0.06	1.84±0.05	1.83±0.05	1.84±0.06	1.89±0.07	1.77±0.06
	Sound	Small	1.84±0.10	1.72±0.09	2.23±0.12	1.82±0.11	1.59±0.07	1.67±0.09
	Control	Small	1.85±0.10	1.68±0.08	1.81±0.14	1.76±0.09	1.72±0.11	1.68±0.10
250	Sound	Large	1.61±0.08	1.69±0.06	1.70±0.10	1.86±0.08	1.78±0.09	1.63±0.06
	Control	Large	1.56±0.08	1.64±0.08	1.61±0.07	1.73±0.09	1.89±0.11	1.52±0.06
	Sound	Small	1.70±0.09	1.70±0.08	1.87±0.19	1.88±0.11	1.67±0.07	1.73±0.11
	Control	Small	1.62 ±0.06	1.70±0.09	1.63±0.14	1.81±0.15	1.66±0.08	1.76±0.13

Small (400 to 600 g) and large (1000 to 1200 g) terrapins were exposed to playback recordings of approaching recreational boats during sound trials and no sound during control trials. Swimming speed was measured for each terrapin every 10 m through the 60 m experimental canal and standardized by body length. One of four playback recordings was started when each terrapin was 10 m from the underwater speaker.

**Table 2:** Summary statistics for terrapin behavioral response to playback recordings of boat engine sounds.

	F-value		d.f.	P-value
**Motor Size (hp)**	**Mean Swimming Speed (body lengths/s)**			
9.9	7.92	4,9		0.76
25	4.14	4,9		0.56
110	1.49	4,9		0.14
250	2.08	4,9		0.74
	**Mean Swimming Depth (m)**			
9.9	2.48	4,11		0.81
25	1.42	4,11		0.54
110	1.02	4,11		0.21
250	1.23	4,11		0.74
	**Mean Change in Pitch (°)**			
9.9	1.61	4,6		0.29
25	1.25	4,6		0.70
110	0.72	4,6		0.17
250	1.89	4,6		0.07
	**Mean Change in Roll (°)**			
9.9	2.31	4,6		0.61
25	0.29	4,6		0.49
110	0.05	4,6		0.73
250	0.11	4,6		0.39

Diamondback terrapin behavioral response variables (swimming speed, swimming depth, change in pitch, and change in roll) were tested in a mixed effects model to see if behaviors changed between times when sound was being played and times when sound was absent. Terrapins did not significantly change any behavior in response to playback recordings.

**Table 3:** Mean swimming depth of diamondback terrapins in response to boat engine sounds.

			Mean Swimming Depth (m)±1 SE							
Motor Size (hp)	Trial Type	Body Size	15 s	30 s	45 s	60 s	75 s	90 s	105 s	120 s
9.9	Sound	Large	0.16±0.01	0.16±0.01	0.13±0.01	0.13±0.01	0.12±0.01	0.13±0.01	0.14±0.01	0.15±0.01
	Control	Large	0.14±0.01	0.13±0.01	0.14±0.01	0.13±0.01	0.13±0.01	0.15±0.01	0.15±0.01	0.14±0.01
	Sound	Small	0.12±0.01	0.13±0.01	0.14±0.01	0.12±0.01	0.11±0.01	0.14±0.01	0.13±0.01	0.14±0.01
	Control	Small	0.11±0.01	0.13±0.01	0.13±0.01	0.12±0.01	0.12±0.01	0.14±0.01	0.13±0.01	0.13±0.01
25	Sound	Large	0.23±0.02	0.24±0.02	0.23±0.02	0.24±0.02	0.23±0.02	0.23±0.02	0.24±0.02	0.23±0.02
	Control	Large	0.23±0.02	0.23±0.02	0.21±0.02	0.21±0.02	0.23±0.02	0.22±0.02	0.23±0.02	0.23±0.02
	Sound	Small	0.21±0.02	0.21±0.02	0.22±0.02	0.22±0.02	0.22±0.02	0.23±0.02	0.22±0.01	0.23±0.02
	Control	Small	0.24±0.02	0.23±0.02	0.23±0.02	0.22±0.02	0.22±0.02	0.23±0.02	0.25±0.02	0.23±0.02
110	Sound	Large	0.24±0.02	0.25±0.01	0.25±0.02	0.24±0.02	0.24±0.02	0.25±0.01	0.24±0.01	0.25±0.01
	Control	Large	0.24±0.02	0.25±0.02	0.25±0.02	0.24±0.02	0.25±0.02	0.24±0.02	0.23±0.02	0.24±0.02
	Sound	Small	0.21±0.01	0.22±0.01	0.23±0.01	0.22±0.01	0.20±0.01	0.21±0.01	0.23±0.02	0.22±0.01
	Control	Small	0.20±0.02	0.19±0.02	0.21±0.02	0.22±0.02	0.20±0.02	0.21±0.02	0.20±0.02	0.19±0.02
250	Sound	Large	0.18±0.02	0.18±0.02	0.17±0.02	0.18±0.02	0.16±0.02	0.17±0.02	0.19±0.02	0.17±0.02
	Control	Large	0.18±0.02	0.19±0.02	0.17±0.02	0.18±0.02	0.17±0.02	0.15±0.02	0.18±0.02	0.17±0.02
	Sound	Small	0.15±0.01	0.15±0.01	0.16±0.02	0.14±0.01	0.16±0.01	0.15±0.01	0.14±0.01	0.16±0.01
	Control	Small	0.15±0.01	0.15±0.01	0.16±0.01	0.15±0.01	0.17±0.02	0.15±0.01	0.15±0.01	0.15±0.01

= 80) was measured before, during, and after exposure to playback recordings of boat engine sounds using a milli-L temperature and depth data logger. Terrapins did not behaviorally respond to boat sounds by changing swimming depth. Swimming depth of terrapins (n

**Table 4:** Mean change in orientation of terrapins in the water in response to playback recordings of boat sounds.

			Mean Change in Pitch (°)± 1 SE			Mean Change in Roll (°)± 1 SE		
Motor Size (hp)	Trial Type	Body Size	Before	During	After	Before	During	After
9.9	Sound	Large	5.69±0.59	6.15±0.48	5.49±0.50	6.67±0.98	6.22±0.32	5.97±0.49
	Control	Large	3.62±0.38	3.06±0.14	3.55±0.22	3.44±0.31	3.30±0.09	3.58±0.19
	Sound	Small	4.77±0.37	5.32±0.40	5.54±0.31	5.59±0.25	6.37±0.35	6.24±0.45
	Control	Small	3.67±0.32	3.28±0.21	3.55±0.19	3.95±0.42	3.34±0.20	3.31±0.13
25	Sound	Large	3.45±0.23	3.53±0.19	3.96±0.43	3.51±0.20	3.55±0.13	3.72±0.20
	Control	Large	3.37±0.23	3.55±0.12	3.97±0.29	3.29±0.28	3.31±0.20	3.32±0.14
	Sound	Small	3.69±0.26	3.64±0.34	4.40±0.20	3.82±0.42	3.84±0.24	3.87±0.25
	Control	Small	4.15±0.25	3.78±0.22	3.84±0.36	4.27±0.23	4.17±0.28	3.64±0.39
110	Sound	Large	3.62±0.40	3.85±0.24	4.08±0.27	3.94±0.22	3.73±0.19	4.01±0.20
	Control	Large	3.89±0.26	3.82±0.25	4.04±0.39	4.22±0.38	3.83±0.19	3.55±0.24
	Sound	Small	4.11±0.38	4.00±0.32	4.88±0.41	4.04±0.27	3.87±0.41	4.40±0.57
	Control	Small	4.29±0.37	4.02±0.26	4.70±0.43	4.63±0.49	3.73±0.32	3.88±0.23
250	Sound	Large	4.27±0.59	4.58±0.43	4.46±0.47	4.87±0.54	4.81±0.65	4.42±0.54
	Control	Large	3.13±0.19	3.39±0.22	2.98±0.30	3.36±0.44	3.20±0.25	3.08±0.22
	Sound	Small	4.02±0.40	4.58±0.36	4.28±0.28	4.34±0.40	4.00±0.27	4.35±0.45
	Control	Small	4.06±0.28	4.30±0.45	4.24±0.39	3.79±0.32	3.82±0.23	3.79±0.32

= 80) before, during, and after exposure to boat engine sounds were calculated to determine if terrapins were changing amount of sudden or erratic movements. Mean change in pitch (x-axis) and roll (y-axis) of terrapins (n

## Discussion

Diamondback terrapins do not behaviorally respond to playback recordings of boat engine sounds and have high rates of boat injuries suggesting that conservation measures are needed to protect viability of terrapin populations. McGregor [28] identified three reasons why significant differences in behavior are not found during playback experiments. First, the subjects may not be able to perceive the difference between control and experimental exposure. In our study, this is unlikely because terrapins physiologically respond to low-frequency sounds less than 1000 Hz [8] and recreational boat engines produce low-frequency sounds within the hearing range of terrapins. Second, the variables measured may not be sensitive enough to detect a significant behavioral response [28]. This is unlikely because the depth and orientation of each terrapin were recorded every 1 s during experimental trials. Swimming speed was measured in each 10 m segment of the experimental canal through which the terrapin swam. It is possible that this variable was not sensitive enough to detect a startle response because terrapins may accelerate at shorter distances than 10 m. Third, animals may be able to detect treatment differences but their behavioral response may be the same regardless of whether or not the sound is played. Because terrapins in Barnegat Bay are exposed to high levels of recreational boating traffic [29], those used in this study may have been habituated to the sounds produced by boat engines and therefore may not respond behaviorally. Further testing using naïve terrapins may determine whether this was the case. Loggerhead and green sea turtles do not behaviorally respond to anthropogenic sounds unless the SPL of the sound exceeds 166 dB re 1 µPa rms [23]. We did not expose diamondback terrapins to boat sounds with SPLs as high as this, but we did not measure boat sounds with SPLs that high in Barnegat Bay. Nevertheless, diamondback terrapins in Barnegat Bay do not respond to boat engine sounds at SPLs that are present in their environment and thus are at risk of injury.

Boat injuries were found in 11% of captured terrapins. This high injury rate only included terrapins that survived their injuries. Thus our estimates of injury rates are conservative because killed terrapins would not be included. We have observed dead terrapins washed up on nesting sites that had major carapace and plastron damage consistent with boat propeller strikes. These observations are consistent with our findings that terrapins do not significantly alter their behavior in response to sounds of approaching boats.

Anthropogenic mortality has an important negative impact on freshwater turtles [30] and sea turtles [31], and can drive populations towards extinction [32]–[34]. The lack of behavioral response of terrapins to the sounds of approaching boats and the probable lack of awareness of boaters to terrapins likely explain the high rate of injuries to terrapins due to boat strikes in Barnegat Bay and other North American estuaries.

Turtles may use cues other than sound to alert them to anthropogenic disturbances such as oncoming boats. For example, terrapins may be able to determine whether a boat is approaching by the shadow cast into the water by the vessel or the displacement of the water by the approaching boat. Harrison [35] found that medium (833 to 895 g) and large (1067 to 1170 g) female terrapins increased their depth in water by 0.16 to 0.18 m respectively, when confronted with a moving boat. However, this change in swimming depth is likely not great enough for a terrapin to avoid contact with a boat propeller.

Boat operators in Barnegat Bay and elsewhere generally do not respond to the presence of terrapins. Boaters speeding through the narrow creeks of the Forsythe National Wildlife Refuge on jet skis or motorized boats appear unaware of terrapins. The majority of boats we saw in the creeks of Forsythe Refuge during the summer months from 2008 to 2010 were personal watercrafts (PWC), small recreational boats with motors up to 150 hp, and small commercial crabbing vessels. Many boaters we observed speed through saltmarsh creeks despite posted speed limits and could easily hit terrapins because water depth is less than 0.5 m deep during low tide. From conversations, some local boat operators are not even aware that terrapins are found in Barnegat Bay.

Terrapins may also travel in the open waters of Barnegat Bay [36] where they are exposed to various boat types from sail boats to speed boats with high powered engines that attain high speeds. In the open waters of the Bay, boaters moving at moderate speeds would not likely see a terrapin in the turbid water ahead. The potential for injury to terrapins was likely very high in both tidal creeks and open bay.

Diamondback terrapin populations throughout the U.S. are threatened by anthropogenic factors including drowning in crab pots [16], [37]–[39], mortality by automobiles while searching for nesting habitat [40], habitat destruction [16], and predation by non-native predators [41]. The impacts of injury and mortality of aquatic turtles caused by boats are an additional significant threat to the viability of terrapin populations. Because terrapins do not react behaviorally to boat sounds and show limited avoidance of fast approaching boats [35], solutions to reducing anthropogenic injury and mortality must include changes to how boats are operated. We recommend that boat operators are educated to how wildlife may be impacted by boats because all states with terrapin populations require a boater education course to obtain licenses to operate a power boat in the US. Information on the ecology and behavior of terrapins and other aquatic wildlife could be included in such courses. This educational component could succeed if reinforced with regulations closing certain areas of terrapin habitat to boats in areas of high terrapin densities during their activity period. In Barnegat Bay, areas of high densities include aquatic habitat adjacent to nesting beaches during nesting season (late May to mid-July). Further research is necessary to determine other high terrapin density areas such as where mating aggregations are located. Boat wakes may also cause shoreline erosion, especially within sandy nesting beaches [42]. By closing areas of high terrapin density to boating, terrapins would be protected from injury and nesting habitat protected from shoreline erosion. Implementing regulations regarding motorized boat use in habitats with high turtle densities and educating boaters about impacts of boats to aquatic wildlife will be beneficial for conserving turtle populations worldwide.
